# Definition and extraction of 2D shape indices of intracranial aneurysm necks for rupture risk assessment

**DOI:** 10.1007/s11548-021-02469-z

**Published:** 2021-08-18

**Authors:** Sarah Mittenentzwei, Oliver Beuing, Belal Neyazi, I. Erol Sandalcioglu, Naomi Larsen, Bernhard Preim, Sylvia Saalfeld

**Affiliations:** 1 Faculty of Computer Science, Otto-von-Guericke University Magdeburg, Universitätsplatz 2, 39106 Magdeburg, Germany; 2 AMEOS Hospital Bernburg, Kustrenaer Str. 98, 06406 Bernburg, Germany; 3 Department of Neurosurgery, University Hospital Magdeburg, Leipziger Str. 44, 39120 Magdeburg, Germany; 4 University Hospital Schleswig-Holstein Campus Kiel, Arnold-Heller-Straße 3, 24105 Kiel, Germany; 5 Forschungscampus STIMULATE, Magdeburg, Germany

**Keywords:** Intracranial aneurysm, Neck curve, Ostium shape, Rupture risk assessment

## Abstract

**Purpose:**

Intracranial aneurysms are local dilations of brain vessels. Their rupture, as well as their treatment, is associated with high risk of morbidity and mortality. In this work, we propose shape indices for aneurysm ostia for the rupture risk assessment of intracranial aneurysms.

**Methods:**

We analyzed 84 middle cerebral artery bifurcation aneurysms (27 ruptured and 57 unruptured) and their ostia, with respect to their size and shape. We extracted 3D models of the aneurysms and vascular trees. A semi-automatic approach was used to separate the aneurysm from its parent vessel and to reconstruct the ostium. We used known indices to quantitatively describe the aneurysms. For the ostium, we present new shape indices: the 2D Undulation Index (UI$_\mathrm{2D}$), the 2D Ellipticity Index (EI$_\mathrm{2D}$) and the 2D Noncircularity Index (NCI$_\mathrm{2D}$). Results were analyzed using the Student *t* test, the Mann–Whitney *U* test and a correlation analysis between indices of the aneurysms and their ostia.

**Results:**

Of the indices, none was significantly associated with rupture status. Most aneurysms have an NCI$_\mathrm{2D}$ below 0.2. Of the aneurysms that have an NCI$_\mathrm{2D}$ above 0.5, only one is ruptured, which indicates that ruptured aneurysms often have a circular-shaped ostium. Furthermore, the ostia of ruptured aneurysms tend to have a smaller area, which is also correlated with the aneurysm’s size. While also other variables were significantly correlated, strong linear correlations can only be seen between the area of the ostium with the aneurysm’s volume and surface.

**Conclusion:**

The proposed shape indices open up new possibilities to quantitatively describe and compare ostia, which can be beneficial for rupture risk assessment and subsequent treatment decision. Additionally, this work shows that the ostium area and the size of the aneurysm are correlated. Further longitudinal studies are necessary to analyze whether stable and unstable aneurysms can be distinguished by their ostia.

## Introduction

Intracranial aneurysms (IAs) are pathological dilations of the cerebral blood vessels. Such a dilation takes place locally and leads to a bulging of the vessel wall. Aneurysms can rupture, leading to hemorrhage in the brain. Approximately half of the ruptures are fatal, and one-third of surviving patients suffer long-term from neurological or cognitive deficits [27]. Approximately 20% of patients carrying IAs have multiple intracranial aneurysms [20].

**Fig. 1:**
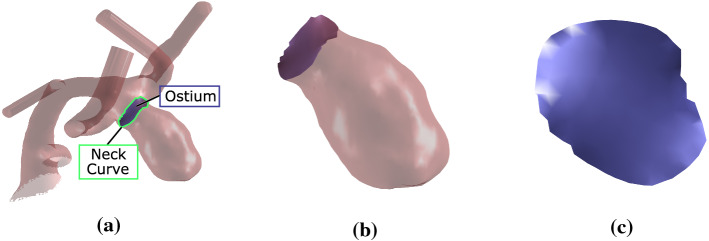
In **a** the parent vessel (light red), the neck curve (green) and the ostium (blue) are depicted. In **b** the aneurysm got separated from the vessel at its neck curve. In **c** the reconstructed ostium is shown

Localization, internal blood flow and geometry of an aneurysm are important indicators for the risk of rupture [12, 25, 28]. Different indices were developed to characterize the shape of the aneurysm sac [30]. Ratios between the aneurysm height or volume and its neck size are also calculated [34]. Quantitative descriptions of the size and shape of an aneurysm enable the comparison of different cases. In the best case, these descriptors can be used to differentiate sets of aneurysms with low and high risk of rupture. The latter may give an indication of the urgency of treatment. In contrast, only size indices like the circumference and area are determined for the neck curve. However, the shape of the neck curve can have a strong impact on the course of treatment and chance of recurrence [33]. There are multiple methods of treatment for IAs, some of which, such as coiling, require precise measurement of the aneurysm’s neck curve to plan the intervention [10].

By segmenting a 3D model of the vessel, the aneurysm can be extracted and the neck curve reconstructed [35]. The 3D triangulation of the neck curve forms the ostium, i.e., the area where the blood flows from the parent vessel into the pathologic dilatation. The calculation of indices using the 3D model is more reliable, since when viewing the 2D image slices or 2D angiographic projections, the perceived size of the neck curve may vary depending on the projection angle or interobserver variability [31, 37].

Despite all efforts, existing features do not suffice to reliably differentiate between aneurysms with a tendency to rupture and safe ones. In this work, we want to propose descriptors for the shape of the ostium and compare quantitative size and shape indices of ruptured and unruptured IAs and their ostia.

## Materials and methods

### Intracranial aneurysm selection and surface mesh extraction

For this study, we analyzed our intracranial aneurysm database comprising approximately 300 patient datasets acquired in daily clinical practice as well as the Aneurisk repository [2]. Due to the large influence of the aneurysm localization on rupture risk and other properties [13, 19], we chose a subset of aneurysms at the middle cerebral artery (MCA) bifurcation with known rupture state. Intracranial aneurysms most often occur at the anterior communicating artery, the internal carotid artery and the MCA [24], whereas our database provided the largest subset for the last category. As a result, we prepared 84 MCA bifurcation aneurysms, of which 27 were ruptured and 57 were unruptured.

For the extraction of the 3D surface meshes of the aneurysm and the parent vessel including the aneurysm’s neck curve, we used the previously described approach [35]. Hence, the vessel’s centerline is employed for semi-automatic extraction of the neck curve that virtually separates the aneurysm from the parent vessel. The centerlines were extracted with the vascular modeling toolkit [3]. Next, we used the neck curves to separate the aneurysm from the parent vessel. Afterwards, we triangulate the neck curve to reconstruct the ostium, see Fig. 1. Based on the extracted surface meshes of the aneurysm sacs and the ostia, we can compare them and automatically extract parameters that quantitatively describe them.

### Parameter extraction based on intracranial aneurysm neck curves

We developed an application to automatically calculate and display different size- and shape-describing indices using MATLAB 2020a (MathWorks, Natick, USA). Size indices are defined as size-related descriptors of the morphology, while shape indices only refer to size-invariant parameters that focus on ellipticity and concavity. All indices are rotation-independent. For the separated aneurysm sacs, volume and surface were calculated as size indices. We used the Undulation Index (UI), Ellipticity Index (EI) and Nonsphericity Index (NSI) as defined in [30] as shape indices. The ostia were projected onto a 2D plane using a principal component analysis. Based on this, we calculated the area and circumference of each projection and its convex hull as size indices. We used the shape indices defined by Raghavan et al. [30] and adapted them to work with 2D shapes:

- The 2D Undulation Index UI$_\mathrm{2D}$,
- The 2D Ellipticity Index EI$_\mathrm{2D}$, and
- The 2D Noncircularity Index NCI$_\mathrm{2D}$.

They are explained in the following and presented in Table 1.

**Table 1:**
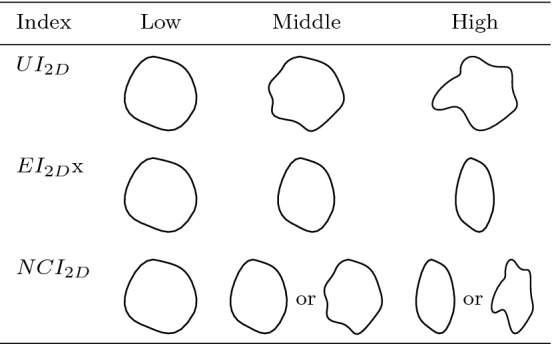
Depiction of the shape indices UI$_{2D}$, EI$_{2D}$ and NCI$_{2D}$

The 2D Undulation Index (UI$_\mathrm{2D}$) calculates the concavity of the ostium border. It is calculated from the area of the ostium *A* and the area of the convex hull of the ostium $A_\mathrm{ch}$. An UI$_\mathrm{2D}$ of 0 represents a convex shape of the ostium. The larger the result, the greater the curvature and therefore also the concavity on the ostium border.

1 $$\begin{aligned} {\text {UI}}_\mathrm{2D} = 1-(A/A_\mathrm{ch}) \end{aligned}$$

The 2D Ellipticity Index (EI$_\mathrm{2D}$) is a measure for how well the ostium may be fitted to an ellipse. $A_\mathrm{ch}$ describes the area and $C_\mathrm{ch}$ the circumference of the convex hull of the aneurysm. The convex hull is used for the calculation to avoid that undulations of the shape influence the index. The EI$_\mathrm{2D}$ varies from 0 to 1, being 0 for a perfect circle and increasing with growing ellipticity.

2 $$\begin{aligned} {\text {EI}}_\mathrm{2D} = 1-2\sqrt{\Pi }\frac{A_\mathrm{ch}^{\frac{1}{2}}}{C_\mathrm{ch}} \end{aligned}$$

The 2D Noncircularity Index (NCI$_\mathrm{2D}$) is a measure of the deviation of the ostium’s shape from a perfect circle. It is calculated similar to the EI$_\mathrm{2D}$, but uses the original area *A* and circumference *C* of the ostium.

3 $$\begin{aligned} {\text {NCI}}_\mathrm{2D} = 1-2\sqrt{\Pi }\frac{A^{\frac{1}{2}}}{C} \end{aligned}$$

Ostia whose index values are close to zero have the approximate shape of a circle, while larger values indicate strongly elliptical or concave shapes, see Fig. 4.

### Statistical analysis

We performed a statistical analysis of the resulting shape index values to assess their predictive power regarding the aneurysm rupture risk based on statistical comparison between ruptured and unruptured aneurysm group. Therefore, we used the two-tailed independent Student *t* tests with a significance level of $p=0.05$. Since the values of the NCI$_\mathrm{2D}$ are not normally distributed, we used the Mann–Whitney *U* test for this variable [15]. The false discovery rate correction was applied to the significant *p* value [6].

In addition, the parameters of the ostium and aneurysm are tested regarding possible correlations between shape of the aneurysm sac and its ostium, using the Pearson correlation coefficient [1]. In our search for correlations, we aim to analyze how size and shape indices of the aneurysm sac and ostium influence each other.

## Results

### Size and shape indices with respect to rupture state

In total, 84 MCA bifurcation aneurysms were used, including 27 ruptured and 57 unruptured cases. There are no significant results for the size and shape indices for the aneurysm sacs. Of our derived indices for the ostium, only the NCI$_\mathrm{2D}$ was significantly associated with the aneurysm’s rupture status at first, see Table 2. However, the false discovery rate correction yields a *p* value for the NCI$_\mathrm{2D}$ of 0.2368, which is no longer below the significance level of 0.05.

**Fig. 2:**
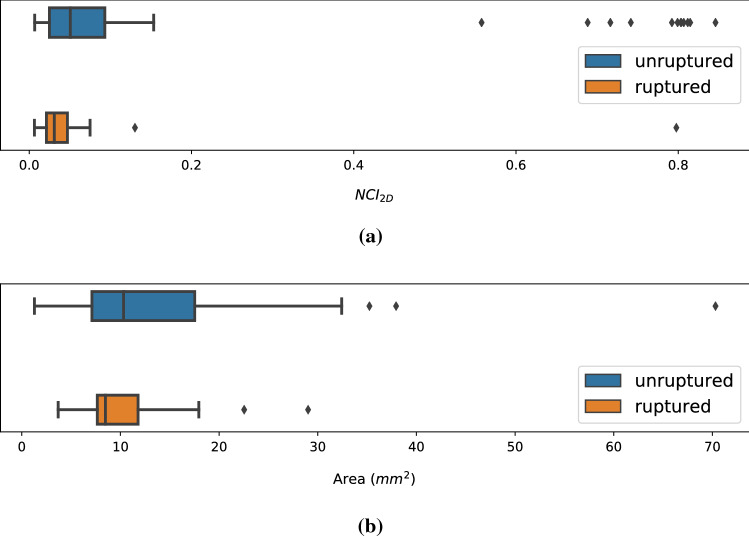
On top a shows **a** comparison of the distribution of $\mathrm{NCI}_\mathrm{2D}$ values for ruptured and unruptured cases as boxplots. Below, **b** shows the distribution of ostium area values for ruptured and unruptured cases

More than 80% of the ostia, both ruptured and unruptured, had small values between 0 and 0.2. Eleven ostia had an NCI$_\mathrm{2D}$ above 0.5, of which only one belonged to a ruptured aneurysm, see Fig. 2a.

**Table 2:** Results of the statistical analysis regarding the aneurysm’s rupture status

Index	*p* value
Ostium area	0.1142
Ostium circumference	0.0652
UI$_\mathrm{2D}$	0.3810
EI$_\mathrm{2D}$	0.7624
NCI$_\mathrm{2D}$	0.0215*
Aneurysm volume	0.9978
Aneurysm surface	0.7466
UI	0.6734
EI	0.7844
NSI	0.5549

Since the values of the NCI$_\mathrm{2D}$ are not normally distributed, the Mann–Whitney *U* test was used. The results of the other parameters were calculated using the Student *t* test. Statistically significant results are marked by an asterisk

* indicates *p* values below a significance level of 0.05

### Correlation with aneurysm indices

**Fig. 3:**
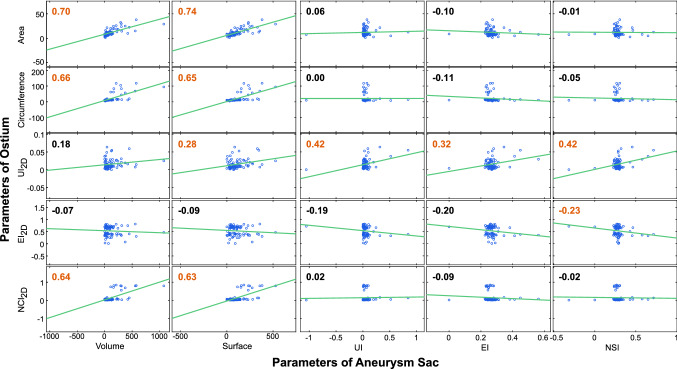
Correlation of the shape parameters of the ostia and the parameters of the aneurysm sacs. The Pearson correlation coefficient is provided at the top left corner of each diagram, and significant correlations are highlighted in red

Furthermore, we compared the parameters of the ostium with the parameters describing the aneurysm w.r.t. a possible correlation using the Pearson correlation coefficient, see Fig. 3. Even though several variables were statistically significantly correlated ($p \le 0.05$), the only strong linear correlation can be seen between the area of the ostium and the volume and surface of the aneurysm. Therefore, ostia with increasing area indicate a corresponding increase in the size of the aneurysm sacs. Our data also show that aneurysms with a small ostium are more frequently ruptured, see examples in Fig. 2b. Thus, small aneurysms are more frequently ruptured in our dataset. However, neither aneurysm size nor ostium area was significantly related to aneurysm rupture status, see Table 2.

## Discussion

The aneurysm size is an often used parameter for assessing the rupture risk of an aneurysm [14]. At the same time, many aneurysms that rupture are small [4, 16]. Many other parameters, such as localization of the aneurysm and parameters describing the internal blood flow, also play important roles [13]. The PHASES score was developed to calculate the rupture risk of an aneurysm within the next five years based on easily obtainable parameters, like age, hypertension and population [17]. However, for multiple aneurysms, the PHASES score is not sufficient, since it might severely underestimate the rupture risk, as only the largest aneurysm contributes to rupture risk evaluation. Furthermore, an external evaluation has shown that the PHASES score results in low specificity for the classification of ruptured and unruptured as well as high-risk and low-risk aneurysms [7].

These data give valuable hints about rupture-prone aneurysms. Research is being conducted to find further simple and reliable parameters. Thus, many studies in the past years focused on 3D reconstructions of the aneurysm sac, its size and other shape indices. There are also various methods for extracting the ostium [21, 26, 35], but it is usually examined only in terms of its size, even though the additional calculation of the shape of an already extracted ostium does not represent a considerable additional effort.

We propose shape indices to reliably describe morphological features of the ostium that are difficult to obtain manually. These shape descriptors allow for a standardized specification and comparison of the ostia. To distinguish between different aspects of the shape, we presented three indices based on generally known indices for the description of the aneurysm sac. The UI$_\mathrm{2D}$ is a measure for the concavity of the ostium’s border, while the EI$_\mathrm{2D}$ calculates the ellipticity of the ostium. The NCI$_\mathrm{2D}$ combines the two previously described indices and measures whether an ostium is rather circular and convex or elliptical and concave.

The neck shape of an aneurysm is considered important when deciding about treatment methods [18]. Currently, treatment decisions are mostly based on the size of the aneurysm and its neck. Large aneurysms (diameter > 10 mm) or wide-necked aneurysms (neck diameter $$ \ge \ $$ 4 mm) are often said to be uncoilable [22]. Studies have shown that their treatment using coils has low occlusion rates and high recurrence rates [5]. Therefore, additional techniques like flow diversion and intrasaccular flow disruption are mainly used for the treatment of large and wide-necked aneurysms. However, these techniques are still assumed to be influenced by the morphology of the aneurysm neck. High neck ratios could lead to lower occlusion rates [29], while a large ostium might cause longer occlusion times [36]. The exact shape of the ostium is not considered in these studies. Therefore, further studies examining the relationship of the proposed indices and occlusion rates as well as recurrence rates should be performed. They might lead to more accurate predictions of the treatment outcome and thus support choosing the appropriate treatment methods.

Besides the ostium indices, we calculated the volume, surface, UI, EI and NSI for each aneurysm sac. In our statistical analysis, no ostium index was significantly associated with rupture status. Of the ostia with an NCI$_\mathrm{2D}$ above 0.5, only one belonged to a ruptured aneurysm. This may indicate a higher rupture probability of MCA bifurcation aneurysms with circular ostia, see Fig. 2a. Unlike in the study of Raghavan et al. [30], none of the shape-describing indices for the aneurysm sac have shown to be significant indicators for the rupture risk. One possible explanation for this is the localization of the aneurysms. Raghavan et al. used cerebral aneurysms of different localization, whereas in our work only MCA bifurcation aneurysms were considered.

Although the area and circumference of the ostium were not significantly associated with the aneurysm’s rupture status, we observed that ruptured MCA aneurysms of our dataset exhibit smaller ostium areas than the unruptured ones, see also the examples in Fig. 2b. Given the fact that ostium area exhibits a strong linear correlation with the volume and surface, and the presence of smaller ostium areas of the ruptured aneurysms, we think the theory that larger aneurysms have a higher risk of rupture than smaller ones, an assumption that is also used in the calculation of the PHASES score, might not be the best solution for this complex relationship. As shown in Table 2, the analysis of the ostium area w.r.t. rupture status got smaller *p* values than the aneurysm volume and the aneurysm surface. We think this analysis could reveal an important trend, and we assume that the ostium area might have a larger influence on aneurysm rupture risk than the evaluation of the aneurysm size without considering the ostium area. This trend should be analyzed in future work for various aneurysm locations. The indices presented are dependent variables, but more independent variables could also be tested in the future. However, the problem of multiple comparisons should be counteracted in this case.

**Fig. 4:**
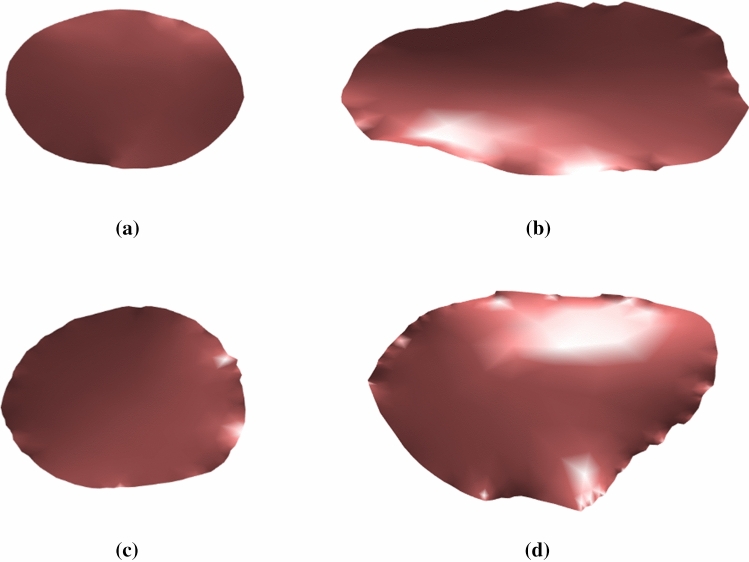
On top ostia from two ruptured aneurysms with **a** small shape indices and **b** large indices are shown. Below are ostia from two unruptured aneurysms with **c** small and **d** large shape indices

In addition to the morphology of the ostium, the blood flow in the aneurysm neck is also of interest for treatment methods, e.g., flow diverters [23]. However, it is complex to calculate, and therefore, not used in clinical practice beyond study. Further studies may use the presented indices to investigate a relationship between ostium shapes and flow patterns. If the shape of the ostium can be used to infer certain flow patterns, this can be a great advantage for treatment decisions.

None of the presented indices were significantly associated with the aneurysm’s rupture status in the presented dataset and therefore do not allow a clear distinction of ruptured from non-ruptured aneurysms. However, they do provide new clues about the nature of the aneurysms and allow a further quantitative description and comparison of different cases. It is difficult or even impossible to distinguish ostia of ruptured and unruptured aneurysms qualitatively, since both classes contain ostia of various shapes, see Fig. 4, and it is hard to estimate which one is more concave or elongated only by viewing the images of the 2D shapes. Therefore, the shape indices give a more reliable rating of concavity and ellipticity. Thus, they can be supportive in the evaluation of aneurysms.

Furthermore, the division into ruptured and unruptured aneurysms is not optimal, as theoretically any aneurysm could rupture at some point in the future. Also, the aneurysms might grow and change their morphological shape. Therefore, some studies used the terms *stable* and *unstable aneurysms*, where stable aneurysms are defined as unruptured aneurysms that have not grown in size by more than 1.0 mm for at least 12 months [9]. Longitudinal studies show that the shape of the aneurysm sac of many unstable aneurysms changes over time [8]. Particularly, the NSI often differs between stable and growing aneurysms [11]. Similar to our results, in the longitudinal study of Ramachandr et al. [32] the NSI was not significantly different between stable and unstable aneurysms. They presumed that the differences between the studies are due to a selection bias. However, the necessary follow-up information required to make a distinction between stable and unstable is not available for our data set, since they were acquired within the clinical routine. Nevertheless, the parameter can influence upcoming clinical decisions. As can be seen from the correlation analysis, an increase in the size of the aneurysm can also be used to infer a change in the ostium and vice versa, see Fig. 3.

The presented morphological analysis of the ostium is embedded into a MATLAB software prototype. Thus, it is easily accessible for clinical researchers and it does not require expensive resources, e.g., for blood flow simulations. Based on our discussions with clinical researchers, the usage of MATLAB-based software prototypes is acceptable for them. On the other hand, this analysis induces additional work load for the clinicians. This is not justified for easy cases or aneurysms with high rupture risk that should be treated immediately, but it might be beneficial for complex cases where a decision must be made as to whether and in what form treatment should be carried out. In addition, the shape parameters could be important for monitoring the aneurysm growth, since they allow for a quantitative comparison of the aneurysm neck over time.

## Conclusion

In this work, we propose indices to quantitatively describe the shape of the ostium. We derived parameters describing the ostia based on commonly used 3D shape parameters: the 2D Undulation Index UI$_\mathrm{2D}$, the 2D Ellipticity Index EI$_\mathrm{2D}$, and the 2D Noncircularity Index (NCI$_\mathrm{2D}$). For statistical evaluation of ostium shape and rupture risk, we evaluated 84 cerebral aneurysms. To account for the dependency of localization and rupture risk, we only considered aneurysms at the middle cerebral aneurysm bifurcation. None of the parameters achieved statistical significance concerning the distinction into ruptured and non-ruptured aneurysms. However, they might have potential for longitudinal analysis since they allow for quantitatively characterizing the aneurysm neck. Based on our correlation analysis, the ostium’s area correlates with the aneurysm sac’s surface area and volume. This might be an indication of the importance of the ostium surface area for future analyses. Finally, the shape descriptors can be beneficial in terms of treatment decisions, since the outcome also depends on the aneurysm’s neck shape.

In future work, the blood flow at the ostium is of interest when choosing a treatment method. Since flow simulations are in most cases too complex to be applied in daily clinical practice, a possible relationship between the shape of the ostium and certain flow patterns would be of interest. For this purpose, flow simulations for the aneurysms could be carried out in further studies. Furthermore, the relation between the presented indices and the outcome of different treatment methods w.r.t. occlusion rates and as recurrence rates could be investigated in the future. This might support the selection of appropriate treatment methods.

Since this work was implemented in MATLAB, which is already used by clinical researchers, an extension of the functionalities is easy to implement.

## References

[1] Akoglu H (2018). Users guide to correlation coefficients. Turk J Emerg Med.

[2] Aneurisk-Team: AneuriskWeb project website, http://ecm2.mathcs.emory.edu/ aneuriskweb. Web Site (2012)

[3] Antiga L, Piccinelli M, Botti L, Ene-Iordache B, Remuzzi A, Steinman DA (2008). An image-based modeling framework for patient-specific computational hemodynamics. Med Biol Eng Comput.

[4] Beck J, Rohde S, el Beltagy M, Zimmermann M, Berkefeld J, Seifert V, Raabe A (2003). Difference in configuration of ruptured and unruptured intracranial aneurysms determined by biplanar digital subtraction angiography. Acta Neurochir.

[5] Becske T, Kallmes DF, Saatci I, McDougall CG, Szikora I, Lanzino G, Moran CJ, Woo HH, Lopes DK, Berez AL, Cher DJ, Siddiqui AH, Levy EI, Albuquerque FC, Fiorella DJ, Berentei Z, Marősfoi M, Cekirge SH, Nelson PK (2013). Pipeline for uncoilable or failed aneurysms: Results from a multicenter clinical trial. Radiology.

[6] Benjamini Y, Hochberg Y (1995). Controlling the false discovery rate—a practical and powerful approach to multiple testing. J R Stat Soc Ser B.

[7] Bijlenga P, Gondar R, Schilling S, Morel S, Hirsch S, Cuony J, Corniola MV, Perren F, Rüfenacht D, Schaller K (2017). PHASES score for the management of intracranial aneurysm. Stroke.

[8] Boussel L, Rayz V, McCulloch C, Martin A, Acevedo-Bolton G, Lawton M, Higashida R, Smith WS, Young WL, Saloner D (2008). Aneurysm growth occurs at region of low wall shear stress. Stroke.

[9] Brinjikji W, Chung BJ, Jimenez C, Putman C, Kallmes DF, Cebral JR (2017). Hemodynamic differences between unstable and stable unruptured aneurysms independent of size and location: a pilot study. J Neurointervent Surg.

[10] Brinjikji W, Cloft H, Kallmes D (2009). Difficult aneurysms for endovascular treatment: Overwide or undertall?. Am J Neuroradiol.

[11] Chien A, Xu M, Yokota H, Scalzo F, Morimoto E, Salamon N (2018). Nonsphericity index and size ratio identify morphologic differences between growing and stable aneurysms in a longitudinal study of 93 cases. Am J Neuroradiol.

[12] Detmer FJ, Chung BJ, Jimenez C, Hamzei-Sichani F, Kallmes D, Putman C, Cebral JR (2018). Associations of hemodynamics, morphology, and patient characteristics with aneurysm rupture stratified by aneurysm location. Neuroradiology.

[13] Detmer FJ, Chung BJ, Mut F, Slawski M, Hamzei-Sichani F, Putman C, Jiménez C, Cebral JR (2018) Development and internal validation of an aneurysm rupture probability model based on patient characteristics and aneurysm location, morphology, and hemodynamics. Int J Comput Assist Radiol Surg 13(11):1767–1779 10.1007/s11548-018-1837-0 PMC6328054 30094777

[14] Dhar S, Tremmel M, Mocco J, Kim M, Yamamoto J, Siddiqui AH, Hopkins LN, Meng H (2008). Morphology parameters for intracranial aneurysm rupture risk assessment. Neurosurgery.

[15] Fay MP, Proschan MA (2010). Wilcoxon–Mann–Whitney or t-test? On assumptions for hypothesis tests and multiple interpretations of decision rules. Stat Surv.

[16] Forget TR, Benitez R, Veznedaroglu E, Sharan A, Mitchell W, Silva M, Rosenwasser RH (2001). A review of size and location of ruptured intracranial aneurysms. Neurosurgery.

[17] Greving JP, Wermer MJH, Brown RD, Morita A, Juvela S, Yonekura M, Ishibashi T, Torner JC, Nakayama T, Rinkel GJE, Algra A (2014). Development of the PHASES score for prediction of risk of rupture of intracranial aneurysms: a pooled analysis of six prospective cohort studies. Lancet Neurol.

[18] Huang CQ, Kang DZ, Yu LH, Zheng SF, Yao PS, Lin YX, Lin ZY (2018). The classification of intracranial aneurysm neck: a single center research experience. Chin Neurosurg J.

[19] Ishibashi T, Murayama Y, Urashima M, Saguchi T, Ebara M, Arakawa H, Irie K, Takao H, Abe T (2008). Unruptured intracranial aneurysms—incidence of rupture and risk factors. Stroke.

[20] Jabbarli R, Dinger TF, Oppong MD, Pierscianek D, Dammann P, Wrede KH, Kaier K, Köhrmann M, Forsting M, Kleinschnitz C, Sure U (2018). Risk factors for and clinical consequences of multiple intracranial aneurysms. Stroke.

[21] Jerman T, Chien A, Pernus F, Likar B, Spiclin Z (2020). Automated cutting plane positioning for intracranial aneurysm quantification. IEEE Trans Biomed Eng.

[22] Jiang B, Paff M, Colby GP, Coon AL, Lin LM (2016). Cerebral aneurysm treatment: modern neurovascular techniques. BMJ.

[23] Karmonik C, Diaz O, Klucznik R, Grossman RG, Zhang YJ, Britz G, Lv N, Huang Q (2014). Quantitative comparison of hemodynamic parameters from steady and transient CFD simulations in cerebral aneurysms with focus on the aneurysm ostium. J NeuroIntervent Surg.

[24] Keedy A (2006). An overview of intracranial aneurysms. McGill J Med MJM.

[25] Kretschmer T (ed) (2017) Zerebrale Aneurysmen und Gefäßmalformationen. Springer, Berlin. 10.1007/978-3-662-50478-9

[26] Meuschke M, Günther T, Wickenhöfer R, Gross M, Preim B, Lawonn K (2018). Management of cerebral aneurysm descriptors based on an automatic ostium extraction. IEEE Comput Gr Appl.

[27] Nadgir R, Yousem DM (2017) Cerebral aneurysms. Requisit Neuroradiol. 10.1002/9781118782934

[28] Niemann U, Berg P, Niemann A, Beuing O, Preim B, Spiliopoulou M, Saalfeld S (2018) Rupture status classification of intracranial aneurysms using morphological parameters. In: 2018 IEEE 31st international symposium on computer-based medical systems (CBMS). IEEE (2018). 10.1109/cbms.2018.00016

[29] Paliwal N, Tutino V, Shallwani H, Beecher J, Damiano R, Shakir H, Atwal G, Fennell V, Natarajan S, Levy E, Siddiqui A, Davies J, Meng H (2019). Ostium ratio and neck ratio could predict the outcome of sidewall intracranial aneurysms treated with flow diverters. Am J Neuroradiol.

[30] Raghavan ML, Ma B, Harbaugh RE (2009). Quantified aneurysm shape and rupture risk. J Neurosurg.

[31] Rajabzadeh-Oghaz H, Varble N, Shallwani H, Tutino VM, Mowla A, Shakir HJ, Vakharia K, Atwal GS, Siddiqui AH, Davies JM, Meng H (2018). Computer-assisted three-dimensional morphology evaluation of intracranial aneurysms. World Neurosurg.

[32] Ramachandran M, Retarekar R, Raghavan ML, Berkowitz B, Dickerhoff B, Correa T, Lin S, Johnson K, Hasan D, Ogilvy C, Rosenwasser R, Torner J, Bogason E, Stapleton CJ, Harbaugh RE (2016). Assessment of image-derived risk factors for natural course of unruptured cerebral aneurysms. J Neurosurg.

[33] Raymond J, Guilbert F, Weill A, Georganos SA, Juravsky L, Lambert A, Lamoureux J, Chagnon M, Roy D (2003). Long-term angiographic recurrences after selective endovascular treatment of aneurysms with detachable coils. Stroke.

[34] Ryu CW, Kwon OK, Koh JS, Kim EJ (2010). Analysis of aneurysm rupture in relation to the geometric indices: aspect ratio, volume, and volume-to-neck ratio. Neuroradiology.

[35] Saalfeld S, Berg P, Niemann A, Luz M, Preim B, Beuing O (2018). Semiautomatic neck curve reconstruction for intracranial aneurysm rupture risk assessment based on morphological parameters. Int J Comput Assist Radiol Surg.

[36] Su T, Reymond P, Brina O, Bouillot P, Machi P, Delattre B, Jin L, Lövblad K, Vargas M (2020). Large neck and strong ostium inflow as the potential causes for delayed occlusion of unruptured sidewall intracranial aneurysms treated by flow diverter. Am J Neuroradiol.

[37] Wong SC, Nawawi O, Ramli N, Kadir KAA (2012). Benefits of 3d rotational DSA compared with 2d DSA in the evaluation of intracranial aneurysm. Acad Radiol.

